# Military-specific application of nutritional supplements: a brief overview

**DOI:** 10.12688/f1000research.6187.1

**Published:** 2015-03-10

**Authors:** Kyle Hoedebecke, Will Brink

**Affiliations:** 1 Department of Family Medicine, Womack Army Medical Center, Fort Bragg, NC, USA; 2 Brink Consulting Group, Natick, MA, 01760, USA

**Keywords:** soldiers, nutrition, supplements, performance, military

## Abstract

The Soldiers of America's military endure numerous physical and mental challenges that demand strict physical fitness regimens, extreme mental agility, and a perpetual readiness to deploy at a moment's notice. The chronicity of these stressors has the potential to dramatically reduce performance - both directly and indirectly.  Because of this risk, many Soldiers turn to nutritional supplements with hopes of optimizing performance. Increasing amounts of research have demonstrated that various supplements may enhance overall physical prowess, health, and offer quicker recovery in the face of corporal or psychological extremes. Most individuals, including many medical and nutrition professionals, possess only an elementary comprehension of nutritional supplements and their effect on Soldiers in training or combat environments. Nevertheless, a grasp of these details is required for safety and optimal benefits. Various compounds have been evaluated - to include evidence within the military setting - and found to augment endurance, increase cognitive function, decrease knee pain, or offer hearing or lung protection in the face of high-energy impulses. These efficacious outcomes may serve to augment the health and longevity of these Soldiers; however, continued research is needed for efficacy and long-term safety within specific environments.

## Introduction

The men and women of America’s military face numerous physical and mental challenges that demand strict physical fitness regimens, extreme mental agility, and a perpetual readiness to deploy at a moment’s notice. The chronic nature of these multiple stressors has the potential to dramatically reduce performance directly or indirectly (see Table 1). Because of this risk, many Soldiers turn to nutritional supplements in an attempt to combat such negative effects. In one instance, the Naval Health Research Center surveyed nutritional supplement usage in the SEAL population and established that nearly 78% of this community uses various nutritional supplements ^1^. Even more perturbing is their reported reliance on fellow operators and bodybuilding magazines for supplement advice – neither of which are considered as accurate or unbiased sources ^1^. Similarly, another study showed that, of 2,215 Americans beginning U.S. Army Special Forces and Ranger training discovered, 85% reported a history of supplement use, 64% stated present use, and 35% stated daily nutritional supplement use secondary to multiple motives ^2^. These findings cohere with the authors’ observations of supplement use in the US Army and it is realistic to believe that corresponding figures may be found in other military branches, though more data is needed for confirmation of such pervasive use.

**Table 1.** Training/combat may negatively impact the following.

Immune suppression
Decreased reaction time
Reduced mental acuity
Increased oxidative stress
Increased susceptibility to heat/cold stress
Blast-induced hearing loss
Blast-induced lung damage
Inadequate nutritional intake

Increasing amounts of research continues to demonstrate that various nutritional supplements may augment performance as well as general wellbeing; at the same time, these compounds must still be targeted to specific cohorts and must complete validation testing for safety and effectiveness. Most individuals, including many medical and nutrition specialists, maintain only a basic grasp of these nutritional supplements and their effects in the face of extreme physical and mental demands of military training and combat. Knowledge of both is required for continued advancement in this field. Though many supplements lack the necessary data to support their use, some existing studies demonstrate compelling and relevant positive benefits. What follows is a brief review of specific nutritional supplements that suggest a positive impact on military populations. Literature search was performed using PubMed and Google Scholar performed in March 2014.

## Protection in the face of high-energy impulse noise

Select groups of Soldiers face regular and repeated exposures to high-energy impulse noise with the potential for permanent hearing loss as well as a decreased operational effectiveness, long-term health decline, and combat readiness ^3^. Multiple studies have found oral magnesium as an efficacious treatment for the prevention of hearing loss secondary to impulse noise. Even before testing in humans, data in animal models, such as guinea pigs, identified direct correlations between decreased serum magnesium levels and harmful noise-induced permanent hearing threshold shifts ^4^ with subsequent evaluations finding similar associations in humans. A placebo-controlled, double-blind study analyzing 300 healthy military recruits undergoing basic training with multiple exposures to impulse noises compared two groups - one receiving 167mg magnesium aspartate orally and the other with placebo daily – with results showing a significant preservation of baseline hearing in the magnesium cohort when compared to the placebo control ^5^. An additional well-performed study confirmed these results demonstrating the protective nature of magnesium for hearing loss secondary to exposure to high impulse noise ^6^. Though it is preferable to have magnesium prior to any exposure, there is evidence that post-exposure oral magnesium supplementation still offers hearing protection with a therapeutic effect inversely proportional to the length of time elapsed between the exposure and the start of treatment ^7^. Its efficacy may be further improved in the presence of other antioxidants – specifically vitamins A, C, and E. These compounds, all of which are commonly found in standard multivitamin tablets, have shown to augment the efficacy of magnesium and its ability to protect against hearing loss ^3^.

Similarly, the lungs also have a heightened susceptibility to these types of injuries secondary to abrupt rises in atmospheric pressure via concussive forces from explosions. Additional low-level exposures are associated with significant pathology within various susceptible parts of the body. A study from the Walter Reed Army Institute of Research showed low-level exposures to correlate with pathological pulmonary changes secondary to oxidative damage caused by antioxidant depletion – increasing the risk of long term problems such as respiratory insufficiency and adult respiratory distress syndrome ^8^. Because of the link to oxidative damage, there is a potential benefit from multivitamin supplementation during military training or deployment when the risk of high impulse energy exposure is at its greatest ^8^. Considering the low cost and risk profile, military medical providers should consider recommending that their Soldiers take multivitamins with antioxidants in deployed or training environments.

## Minimizing musculoskeletal injuries despite prolonged training and operational requirements

The often unimaginable amounts of stress on both mind and body during simulated or live combat situations often correlate with increased injury rates. Joint pain, fractures, and other pathologies plague recruits, trainees, and veteran operators alike – a worrisome situation where individual and unit readiness are potentially sacrificed. Implementing measures to safely minimize or treat injuries would contribute to combating work-related stressors and subsequently augmenting a unit’s operational effectiveness. One such detractor is chronic pain from degenerative joint disease (DJD) caused by and worsened with repetitive microtrauma. Several combinations of glucosamine, chondroitin, and manganese have been effective for this ailment in both dogs and humans ^9– 12^. The Naval Special Warfare Command performed a 16 week randomized, double-blind, placebo-controlled crossover trial with 34 Soldiers suffering from chronic knee or lumbar pain secondary to DJD. Half were placed in a group given glucosamine HCl (1,500 mg/day), chondroitin sulfate (1,200 mg/day), and manganese ascorbate (228 mg/day) while the others received a placebo control. Though not effective for back pain, this supplement combination has proven effective for the treatment of knee pain in this study and multiple other studies ^9, 11– 12^.

Moreover, Soldiers and trainees experience numerous myalgias, contusions, sprains, and other acute injuries caused by high training and operational tempos. More worrisome is the potential reduction in overall health and immunity, increased susceptibility to infections, and higher likelihood of heat-related injuries. One study evaluated such outcomes in relation to post-exercise protein consumption in Marine recruits. These healthy males were randomly assigned to three treatment arms – placebo, control, and protein supplement – during a basic training period of 54 days. Compared with the other 2 arms of the study, the protein supplement cohort averaged 33% fewer total medical visits, 28% fewer bacterial/viral infections, 37% fewer muscle/joint problems, and 83% fewer visits due to heat exhaustion ^13^. Post-exercise protein supplementation, as seen in this study, offers the ability to positively improve medical outcomes, musculoskeletal resiliency, and tissue hydration during extended levels of physical demand – signifying a potential medical therapy allowing for the avoidance of various health problems found in populations undergoing physical and mental extremes ^13^. These outcomes may further have a positive effect by improving recruit/trainee dropout rates, reducing number of sick days, and decreasing overall medical expenditures.

## Mental and physical maintenance under stress

B-alanine (BA) – a known substrate of carnosine – has been shown to augment prolonged physical performance. This supplement plays an important role helping to buffer hydrogen ions during high-intensity exercise ^14^. A meta-analysis of 15 peer-reviewed publications evaluating the effects of BA – with a median of 179 g BA supplemented – on exercise concluded that there was a significant improvement for exercise lasting 60–240 seconds ( *P* = 0.001) as well as >240 s ( *P* = 0.046) compared to placebo ^15^. Of note, there was no improvement for exertion less than 60 seconds ( *P* = 0.312) ^15^. Similar improvements have also been shown in Soldiers. A group of 20 elite operators were placed in either a BA group or placebo group for a 4 week period and had a variety of military and physical fitness tests before and after supplementation. At the end of the study, the Soldiers in the BA arm showed no improvement in cognition; however, they did show significantly better power performance, marksmanship, and target engagement speed from pre-ingestion levels as compared to placebo ^15^.

Caffeine use has also displayed improvement in various aspects of a Soldier’s performance ^16– 17^. A prospective randomized study of 62 trainees during Navy SEAL Hell Week placed the participants into one of four arms: 100, 200, or 300 mg of caffeine or a placebo. The marksmanship of each group was tested at baseline and again after exposure to training stressors and 73 hours of sleep deprivation. The results showed that 200 to 300 mg of caffeine allowed individuals to sight targets and fire faster while maintaining accuracy during periods of high stress and combined sleep deprivation ^17^. Furthermore, a meta-analysis of 41 placebo-controlled double blinded studies found further evidence of caffeine’s ergogenic effects that include improved exertional capabilities, decreased perceived exertion during ruck marches, better reaction time, improved memory and overall cognitive function, better mood, and overall improved performance ^16^. When used in low to moderate doses between 38 to 400 mg caffeine daily, Soldiers and military units can take advantage of the positive operationally pertinent effects of caffeine while minimizing potential side effects such as dehydration or insomnia ^18^.

## Conclusion

This paper is not meant to be an exhaustive review of every supplement with possible direct military applications, but rather to identify the potential of these compounds through the above specific examples which were conducted with the appropriate population. Limitations of this study include a non-systematic review approach and the possible omission of relevant studies – including those with negative or null results. This topic remains a highly controversial subject though data exists illustrating the potential benefit of many nutritional supplements. The authors suggest that much of this dispute stems from the continued need for additional data, exaggerated marketing claims, and a lack of FDA regulation; however, the military medical community is not taking advantage of the potential positive benefits by choosing to ignore virtually all nutritional supplements. Additional research and a streamlined method of training and information sharing among providers are still necessary and potentially beneficial.

Furthermore, various inexpensive supplements may serve to save the military millions of dollars. For each compound, a cost/benefit analysis must be conducted for recommendations of location, longevity, and amount of supplements use. Areas where the military can benefit monetarily in the short term are through reduced recruit/trainee dropout rates or suppressed rates of injury and in the long term through a decline in known associated medical problems (arthritis, hearing loss, traumatic brain injury (TBI), etc.) and reduced turnover rates by increasing a Soldier’s operational effectiveness and longevity.

Utilizing the latest research would allow for the preparation of dedicated formulas within specific military populations based on the likely stressors that each would encounter; however, no relationship exists between the published benefits of certain supplements and the military’s recommendation of their use – leaving the individual Soldier to make do with information supplied by non-vetted sources. Unit medical providers must be knowledgeable in the area so as to inform their Commanders and Soldiers of proper and suggested use. Unfortunately, too many studies and supplements exist for this short article to review adequately. Regardless, the options discussed above have evidence showing a positive effect in lengthening Soldiers’ operational life span and should be considered in a front-line approach.
