# Supplementary material for: Military-specific application of nutritional supplements: a brief overview
Source: F1000Res. 2015 Mar 10;4:61. [Version 1] doi: 10.12688/f1000research.6187.1 (PMC4406188; doi:10.12688/f1000research.6187.1)
Supplement: Peer review history

# Peer review: Referee response for version 1

**DOI:** 10.5256/f1000research.6632.r7905

**Published:** 2015-04-20

**Authors:** Tim N. Ziegenfuss

**Affiliations:** 1 The Center for Applied Health Sciences , Stow, OH, USA

I applaud the authors for their efforts on this paper. The topic is extremely important, and the information is accurate and well written. I only wish they had delved deeper into the related literature as there are many other studies on the supplements they reviewed as well as a vast array of nutritional supplements that have the potential to improve military readiness and/or performance. That said, both of these limitations have been acknowledged by the authors. I encourage them to publish several "follow up" papers to address them.

I have read this submission. I believe that I have an appropriate level of expertise to confirm that it is of an acceptable scientific standard.

# Peer review: Referee response for version 1

**DOI:** 10.5256/f1000research.6632.r7906

**Published:** 2015-03-10

**Authors:** Douglas S. Kalman

**Affiliations:** 1 Nutrition/Endocrinology Department, Miami Research Associates, Miami, FL, USA

The authors did a good job of presenting background, rationale and motivation for their brief overview.

If magnesium aided for protecting hearing or reducing loss of hearing in those exposed to impulse noise, how come there is not much data on use in hearing loss outside those in this setting?

One area that appears overlooked and worthy for inclusion is the aspects of military training, correlation with injury and or illness (e.g., during basic training or elite training). Meaning, research by Flakoll, McClung, Tharion, Etzion-Daniel, Lappe and others appears not covered but yet worthy of at least a paragraph.

Article acceptable with minor edits in that authors add to their writing using the above names and leads.

I have read this submission. I believe that I have an appropriate level of expertise to confirm that it is of an acceptable scientific standard.

# Response

**Published:** 2015-03-10

**Authors:** Will Brink

**Affiliations:** Brink Consulting Group, USA

Dr. Kalman, Thank you for your considered response to our paper. To your Q:

*"If magnesium aided for protecting hearing or reducing loss of hearing in those exposed to impulse noise, how come there is not much data on use in hearing loss outside those in this setting?"*

An excellent question to which we do not have an adequate answer. As you can see, the data is quite supportive and compelling  of the protective effects of magnesium to hearing due to high impulse noise in animal and human models. Seemingly, another example a lack of awareness of the potential benefits of this non toxic and inexpensive mineral that could be of benefit in many industrial settings where hearing damage so common, especially where people are unable to wear hearing protection.

*"One area that appears overlooked and worthy for inclusion is the aspects of military training, correlation with injury and or illness (e.g., during basic training or elite training). Meaning, research by Flakoll, McClung, Tharion, Etzion-Daniel, Lappe and others appears not covered but yet worthy of at least a paragraph."*

We do cover briefly in various locations in the paper, as well as Table 1, some of the negative effects of training and combat on immunity, performance and other effects. Do you feel it's a lack of depth on that particular issue that should be added? Perhaps some objective numbers, via the authors you supplied, on rates of injury and drop out rates.

# Response

**Published:** 2015-03-11

**Authors:** Douglas S. Kalman

**Affiliations:** Miami Research Associates, USA

Links for articles that I think are worthy if possible of a mention:

Vit D - http://www.ncbi.nlm.nih.gov/pubmed/25005834

Iron - http://www.ncbi.nlm.nih.gov/pubmed/24188143

Tyrosine - http://www.ncbi.nlm.nih.gov/pubmed/17078981

An "in-general" study on baseline nutrition of "military" (sub-par to start with) - http://www.ncbi.nlm.nih.gov/pubmed/18849866

Vitamin D - Calcium and military stress fractures: http://www.ncbi.nlm.nih.gov/pubmed/18433305 and

http://www.ncbi.nlm.nih.gov/pubmed/11305081

If any of the above links can be added GREAT, as helpful for the picture you share.

# Response

**Published:** 2015-03-13

**Authors:** Will Brink

**Affiliations:** Brink Consulting Group, USA

Excellent information, thank you. I have some of that data, but we didn't want to make the review overly lengthy but it might make sense to add additional sources. The Tyrosine data has always been of particular interest. A mention of military baseline nutrition being less than optimal would assist the overall message of the review.
